# CONSCIENTIOUSNESS MODERATES THE RELATIONSHIP BETWEEN INTERLEUKIN-6 AND MEMORY DECLINE

**DOI:** 10.1093/geroni/igae098.4145

**Published:** 2024-12-31

**Authors:** Suzanne Segerstrom

**Affiliations:** Oregon State University, Corvallis, Oregon, United States

## Abstract

Evidence supports the role of the pro-inflammatory cytokine interleukin-6 in accelerating cognitive decline and increasing ADRD risk. Personality may influence this relationship in a pathoplastic way, by protecting against or exacerbating the cognitive consequences of inflammation. Conscientious (i.e., controlled, planful, organized) people are characterized by healthier behaviors, which may play a protective role. Among 182 older adults (Mage=74 years, 41% female), Rey Auditory Verbal Learning Test (RAVLT) performance was measured annually for up to 16.6 years (M=6.5 years). Total learning and delayed recall were regressed on expected performance (norms based on age, gender, and IQ), time, IL-6, conscientiousness, and their interactions with each other and with gender. There was a significant interaction among time, IL-6, and conscientiousness (F(1,167.51)=5.36, p=.021) such that people with high conscientiousness and low IL-6 maintained total learning and thus increased their performance relative to norms over time; people with low conscientiousness and/or high IL-6 maintained performance relative to norms. The same interaction was observed for delayed recall (F(1,165.22)=5.20, p=.024). The recall effect decreased after adjusting for learning, but in a gender-specific way (interaction with gender, F(1,164.74)=4.97, p=.027). Adjusting for total learning eliminated the interaction for men but not for women. Rather than a pathoplastic relationship, IL-6 and conscientiousness had a synergistic effect on memory decline over time. Neither low IL-6 nor high conscientiousness alone were sufficient to preserve memory. The healthy habits of conscientious older people may need an advantageous physical environment in order to affect memory and possibly other cognitive functions.

